# Round the Hospitals

**Published:** 1918-03-23

**Authors:** 


### ROUND THE HOSPITALS.

The War Office is again appealing for more nurses with three years' general training, and states that the rate of pay has now been increased for all who have served twelve months. The military needs represent the first call on nurses as they complete their training, and it reflects credit on every training-school to show a high percentage of newly certificated nurses responding to the national call. Insistent as the claims of the wounded may appear to be, and loudly as they resound in every ear, it is not superfluous for matrons and sisters to hold steadily before probationers throughout their pupil years the country's paramount claim to their services when the moment comes.

The "Mere Man," who takes up the cudgels in the *Times* on behalf of sisters, nurses, and V.A.D. workers in France, wakes a responsive echo in many breasts by exposing the grievances under which the Nursing Service labours in regard to leave. It is not that any one grudges working to the utmost limit of their capacity when emergency demands. It is that faulty organisation on the part of individuals is still responsible for much obstruction of an otherwise admirable machine. More relief should be given in slack times, more determination shown to discover and rectify unaccountable omissions. If one nurse in a hundred has a legitimate grievance, the foresight and

☛ Previous articles appeared Dec. 8, 15, 22, Jan. 5, 12, 26, Feb. 2, and March 2 and 9, p. 489.

**ROUND THE HOSPITALS—*(continued)*.**

organising skill which have eased the lot of all her more fortunate sisters will be overlooked, and the public who are in a mood to champion nurses through thick and thin will want to know the reason why.

The question of whether the authorities are justified in prohibiting British Army nurses from attending dances, which is argued in the same letter, is more complicated than the writer guesses. No one in or out of authority believes dancing to be wrong. It is purely a question of expediency. Here is a very large body of (mostly young) women, drawn from every class of society, undertaking in a foreign country duties of the most exacting description. Their efficiency and the good repute of the Nursing Service depend on their ability to surrender their minds entirely to the work they have undertaken. Naturally this demands much sacrifice of inclination. Moreover they work in close association with men of other nationalities, who may imperfectly apprehend their outlook. Reserve and formality are indicated. Does the excitement of the dance make the nurse keener over her duties, or does it accentuate languor and a desire for pleasure? Can the barriers which common sense places between the sexes under existing circumstances be maintained in the ball-room? In so large a company of nurses are there no frivolous ones who might bring reproach on the whole body? The conclusion we reach is that the women who care for our wounded in war are consecrated beings. They work under a great tradition and are content, proud even, to surrender legitimate occasions for gaiety if by so doing they may be a source of added strength to their comrades of either sex.

In commenting on the tragedy of the *Glenart Castle* we called attention to the fact that nurses frequently have relatives dependent on them, and we inquired whether in cases of this description any Government pension was available to relieve the distress caused by the death of the bread-winner. We are glad to note that Sir Archibald Williamson has recently raised the same point in the House of Commons. He asked Mr. Hodge, the Minister of Pensions, whether Army nurses are eligible for pensions if they suffer injury through the war, and, if so, would steps be taken to place them on the same footing as other branches of the Army by making provision for dependents in cases where a nurse loses her life? Mr. Hodge, while stating that Army nurses suffering injury through military service are eligible for pensions, went on to say that no cases of relatives dependent on Army nurses, who had been killed or injured in the service, had been brought to his notice, but he readily consented to consider the question of making such provision should information reach him that it was needed. He would not, however, commit himself on the further question of whether the basis of assessment would be similar to that which obtains for the fighting forces.

The fact is that the public has grown accustomed to the idea that nurses must sacrifice not merely themselves, their leisure, their bodily health, and worldly interests if required, but also their relatives at the call of duty, and do not even yet realise their responsibility towards the women who do noble deeds instead of dreaming about them. The nurse frequently bears up the pillars of her home, and for this cause finds herself forlorn at the end of an apparently prosperous career. When she dies or is disabled under circumstances arising out of her war service her relatives ought to receive precisely the same consideration from the War Office as the relatives of combatants. If no applications for help have reached the Minister of Pensions from relatives dependent on nurses disabled or killed in the war we must attribute this to ignorance on their part of the fact that such claims are likely to be sympathetically dealt with.

At the recent Conference at the Women's Institute on the Future of Nursing, Miss Cox Davies had many interesting things to say about the College of Nursing, and none more significant than that while the College had the support of the leading training-schools, smaller ones wrote and asked how they could bring themselves up to the requirements so as to get their nurses on the Register. The College undoubtedly owes its brilliant initial successes to perception on the part of the founders that in order to improve the conditions of nursing it was necessary first to gain the training-schools. When the list numbering some 120 hospital and Poor-Law infirmary training-schools throughout the kingdom, from which candidates at the last election were accepted, is carefully studied, the hesitating and doubtful cannot fail to be convinced that the required support has been freely and widely accorded.

Sir Arthur Stanley recently alluded to himself as one of the largest employers of nurses in the country. The lately issued Report of the Joint Finance Committee of the British Red Cross Society and the Order of St. John of Jerusalem bears out this statement, for it explains that the system of supplying nurses to auxiliary hospitals has developed enormously during the past year. The Nurses' Department has posted 2,393 nurses to home hospitals and 372 to foreign service, and has dealt with 1,480 fresh applications for service with the Joint Committee. It is impossible to form an estimate of the amount spent on the Red Cross Nursing Service, as each hospital bears its own expenses in this connection. But certain grants have been made which directly benefit members of the Nursing Service. The sum of £5,000 has been expended on the Edith Cavell Homes of Rest, the Overseas Reception Committee have received £228, and Queen Mary's Hostel £7,500.

The Infirmary Committee of the Kingston Board of Guardians have confirmed the appointment of Sir James Cantlie as examiner of probationers, an office which Sir James has held for

**ROUND THE HOSPITALS**—(*continued*).

fourteen years. An attack was recently made on the examiner by one of the Guardians, on the odd ground that "the examination results had been so extremely good that he had become suspicious." The committee went carefully into the question of whether it was desirable to make a change periodically in the examiner. It was found on inquiry that, out of eleven Poor-Law infirmaries round London only two made a change annually, while the other nine adopted the same principle as at Kingston and kept their training-schools under the same examiner from year to year. There is much to be said for the continuity maintained by one influence. If variety is the one desideratum in an examination-paper we opine it is more likely to be attained in successive papers set by one man, on his guard not to repeat himself, than in papers set by different men, all probably selecting salient and similar nursing points for their examinees. But we should like to see an outside nurse examiner chosen to supplement the work of the medical examiner in Poor-Law infirmaries, as is done in some of the best general hospital training-schools.

---

Conditions of health and family affairs are the two factors which play havoc with the nursing staff everywhere, cause interruption to the career of promising probationers, and render the lives of matrons a burden to them. In old days, there is good reason to believe, ill-health was by far the most fertile source of resignations. It appears, however, from statistics given by Lady Ampthill in her interesting speech at Norwich last week, as Director of County Organisations of the Voluntary Aid Detachments, that retirements due to ill-health are only half as numerous as those from the other cause. The actual figures last month show thirty-seven retirements on account of health and seventy for family reasons, no doubt including marriage. It would be interesting to know whether this rate of "unavoidable causes" is borne out by the experience of matrons in the matter of nurses and probationers.

---

At Warwick Assizes the discharged district nurse, who has been libelling her former matron for dismissing her under the instructions of her committee, has been fined £10 and is to be detained in custody till the money is paid. The Judge said the woman was evidently suffering from delusions and there was not a shadow of foundation for her charges. She appears to be of the type, unfortunately only too well-known in the nursing world, ill-balanced to start with (for her conduct "upset the nursing staff"), and unhinged by mental concentration on a supposed grievance. There is no career more likely to prove disastrous to such a woman than nursing. Matrons who find neurotic women of this character among their staff are almost certain to incur their lasting resentment, and the initial mistake of allowing them to enter the Nursing Service is apt to be visited heavily on the shoulders of all subsequent employers. As a manual worker, particularly in the open air, the poor nurse might have led, perhaps will still lead, a useful and happy life.

---

Some discontent has been caused in Halifax by the statement that an average of one pound of meat per week is allowed for each officer and inmate in the local hospital and also in the workhouse. Working people were dissatisfied that the infirm and aged people should receive a larger meat ration than was available for the ordinary man in the street. The workhouse authorities urge in their defence that the bread ration is compulsory in all Government institutions, though only voluntary for the public, and that substitutes for meat, such as fish or cheese, are not at the disposition of the workhouse master in the quantities required for the 500 or 600 people in his charge. There is less choice of vegetables in institutions, and no power to make up with one thing when another is unobtainable. Nor can any reserves be accumulated; these big households must now be fed almost from hand to mouth. Moreover, the pound of meat per head is reckoned by the carcase as it comes from the butcher, and most of it is used in making stews and soup. Thus the apparent anomaly is fully explained.

---

In Dr. Leslie Mackenzie's Report on "Scottish Mothers and Children," recently issued by the Carnegie United Kingdom Trust, there is a remarkable chapter on the "Training of Health Visitors," which marks a new epoch in the development of this branch of the public service and has an important bearing on the training of nurses. Dr. Mackenzie would take his health visitor at the age of seventeen or eighteen, and would expect from her a general education such as training-centres for teachers would require—viz., the possession of an intermediate certificate. He would send her for a year to a school for domestic science, giving her besides practical instruction in the care and feeding of normal children in a well-conducted day nursery. The next stage comprises two years' training (Dr. Mackenzie recognises that three would be better) in an approved hospital, and the final six months of the course is to be given to maternity work and midwifery. The scheme is put forth not as in any way complete but as "a basis for discussion." It is valuable as an attempt to bridge over the time between leaving school and beginning hospital training. It tackles courageously the need for attracting students with a good general education, and it lays down the axiom that hospital training is indispensable. The cost of such a four or five years' training must prove prohibitive to many otherwise suitable candidates, and it is considered essential that help should be available for such as will undertake to complete the whole course. It may be that the first demand for scholarships on the College of Nursing may be to enable students to take the long and specialised training which will be needed by the health visitors of the future.

**ROUND THE HOSPITALS—(*continued*).**

No one earns a better income than the masseuse who has succeeded in building up a good connection under good doctors. No one can earn less than the unsuccessful masseuse, for her gains are a minus figure. Between the two lie a multitude of masseuses whose earnings are of a precarious nature, who are liable to long lean periods which deplete their savings through no fault of their own, and may be said to live on the edge of something like destitution. There has long been a feeling that the Incorporated Society of Trained Masseuses ought to have it in their power to relieve the worst cases of distress among their members, and for this purpose a Members' Fund was recently started. Its trustees are Sir Charles Nicholson, M.P.; Mr. Louis Dick, Secretary of the Royal National Pension for Nurses; and Dr. F. Barrie Lambert; and a small administrative committee has been appointed for the Fund, which ought to command confidence and show a rapid growth.

The French War Emergency Fund, which has since 1914 done splendid work in supplying French military hospitals with stores and comforts, has lately taken on a new branch of work. A group of villages in the re-conquered region of the Somme has been handed over to the Fund by the French Government, and a staff of workers has been specially detailed to this work of mercy. They provide extemporised accommodation for the inhabitants as they drift back to their ruined homes, and supply roofing, garden, and farm tools, clothing, cooking utensils, beds and bedding, food, and farmyard stock.

One of their delegates reports: "We have just returned from a trip taken back of the Front, where we brought aid to a hospital recently bombed by the Germans. Four large wards of one hospital were burned to the ground, and many were seriously damaged. One whole section must begin again from the beginning, including operating-rooms. Their needs, of course, were large." One hospital with accommodation for 4,000 men, with a section for skull and spine cases, possessed but a single pillow, no water-beds, no chair of any kind, no bed-tables, no back-rests. In such conditions it is not surprising to learn that the help promptly sent by the society from its headquarters—44 Lowndes Square, London—evokes the universal cry "Vivent nos alliés qui ont pitié."

☞ It is our invariable practice to pay for all items of news which we may publish. The minimum payment is 5s. Paragraphs of about twenty lines in length—that is to say, of 150 words or less—are preferred. Contributions should be addressed to The Editor, The Hospital, 28 and 29 Southampton Street, Strand, London, W.C. 2, and, to be in time for the current week's issue, should reach him by the first post on Mondays.

☞ For Matrons' and Sisters' Appointments, see p. 538.

